# Inhibition of Ape1 Redox Activity Promotes Odonto/osteogenic Differentiation of Dental Papilla Cells

**DOI:** 10.1038/srep17483

**Published:** 2015-12-07

**Authors:** Tian Chen, Zhi Liu, Wenhua Sun, Jingyu Li, Yan Liang, Xianrui Yang, Yang Xu, Mei Yu, Weidong Tian, Guoqing Chen, Ding Bai

**Affiliations:** 1 National Engineering Laboratory for Oral Regenerative Medicine, West China Hospital of Stomatology, Sichuan University, Chengdu 610041, P.R. China; 2 State Key Laboratory of Oral Diseases, West China Hospital of Stomatology, Sichuan University, Chengdu 610041, P.R. China; 3 Department of Orthodontics, West China School of Stomatology, Sichuan University, Chengdu 610041, P.R. China; 4 Department of Oral and Maxillofacial Surgery, West China School of Stomatology, Sichuan University, Chengdu 610041, P.R. China

## Abstract

Dentinogenesis is the formation of dentin, a substance that forms the majority of teeth, and this process is performed by odontoblasts. Dental papilla cells (DPCs), as the progenitor cells of odontoblasts, undergo the odontogenic differentiation regulated by multiple cytokines and paracrine signal molecules. Ape1 is a perfect paradigm of the function complexity of a biological macromolecule with two major functional regions for DNA repair and redox regulation, respectively. To date, it remains unclear whether Ape1 can regulate the dentinogenesis in DPCs. In the present study, we firstly examed the spatio-temporal expression of Ape1 during tooth germ developmental process, and found the Ape1 expression was initially high and then gradually reduced along with the tooth development. Secondly, the osteo/odontogenic differentiation capacity of DPCs was up-regulated when treated with either Ape1-shRNA or E3330 (a specific inhibitor of the Ape1 redox function), respectively. Moreover, we found that the canonical Wnt signaling pathway was activated in this process, and E3330 reinforced-osteo/odontogenic differentiation capacity was suppressed by Dickkopf1 (DKK1), a potent antagonist of canonical Wnt signaling pathway. Taken together, we for the first time showed that inhibition of Ape1 redox regulation could promote the osteo/odontogenic differentiation capacity of DPCs via canonical Wnt signaling pathway.

Dentin is a highly mineralized tissue which constitutes the bulk of the tooth in humans and many other mammalian species^1^. Importantly, dentin functions primarily to protect pulp tissue inside and be a good support for enamel outside^2^. The formation of dentin, known as dentinogenesis, is initiated by differentiation of dental papilla cells (DPCs) into odontoblasts depending on reciprocal and sequential interactions between dental epithelium and mesenchymal^3^,^4^. Except for the dentin formation, DPCs also possess powerful multi-lineage differentiation capacity. These postnatal stem cells can differentiate into adipocytes, osteoblasts/odontoblasts, chondrocytes, and neurons just like other dental mesenchymal stem cells (MSCs)^5^,^6^,^7^. Under physiological conditions, DPCs are promising candidate for cell-based therapy to treat liver disease^8^. Besides, DPCs can form tubelike structures when they are incubated on Matrigel and differentiate into endothelial and epithelial-like cells, which might manifest a possible contribution to vascularization^9^. Except its outstanding multiple differentiation potency, DPCs are highly proliferative and can be expanded and maintained for nearly 60 population doublings, during which they keep their spindle-shaped morphology^10^,^11^.

During the dentin formation, the differentiation and proliferation of DPCs have been shown to be manipulated by multiple signaling networks composed of numerous molecules^12^,^13^,^14^,^15^. Ape1/Ref-1 (Apurinic apyrimidinic Endonuclease/Redox effector factor 1), also called Apex1 or Ref-1, is a molecule with dual functions in DNA repair^16^,^17^ and redox regulation of gene transcription. Because of its functional pleiotropy, Ape1 plays a central role in regulating the cellular response opposed to oxidative stress and maintaining the genome stability and transcriptional activity^5^. Transcription factors such as Nuclear Factor-kappaB (NF-kB)^18^, cAMP response element binding protein (CREB)^19^, Early growth response protein-1 (Egr-1)^20^, inducible factor-1α (HIF-1α)^21^, P53^22^, activator protein-1 (AP-1)^23^ and others have been reported to be activated by Ape1 dependent redox activation. Notablely, Ape1 has a closely connection with the self-renewal and differentiation of stem cells, and redox regulation of Ape1 acts as a pivot during this process. In cardiac stem cells, Ape1 inhibition followed by H_2_O_2_ treatment resulting in significant elevation in cardiac differentiation and apoptosis^24^. Similarly, the suppression of Ape1 redox function performed by E3330, either to the neurogenic embryonic carcinoma cell line NT2-D1 or to hAT-MASC, increases the differentiation of stem cells towards a neural phenotype, biasing the differentiation towards specific subtypes^25^.

The effect of Ape1 in stem cell differentiation has been widely studied in many fields. However, little knowledge is available about its effect on the differentiation of DPCs. In this study, we hypothesized that Ape1 regulates proliferation and osteo/odontogenic differentiation of DPCs through its redox functional domain. For this purpose, DPCs were isolated from impacted human third molar tooth germ and treated with Ape1-shRNA or E3330 an inhibitor of Ape1 redox regulation. We tracked the expression of Ape1 during tooth germ developmental process and investigated the related signaling pathway. In sum, our experimental data showed that the osteo/odontogenic differentiation of DPCs was enhanced by E3330 and Ape1-shRNA, and canonical Wnt signaling was involved in this process.

## Results

### Temporal and spatial expression of Ape1 during rat tooth development

**Figure 1:**
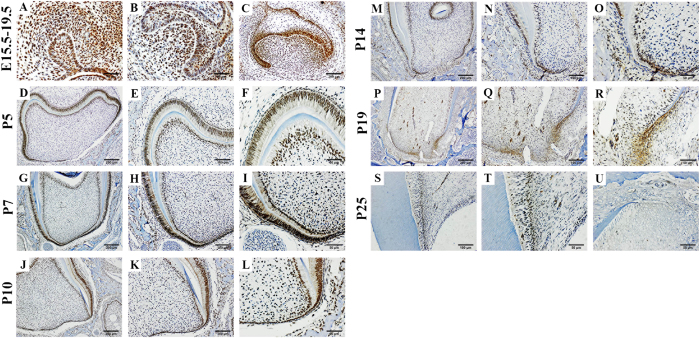
The temporal-spatial expression of Ape1 at different time points. Localization of Ape1 in the mandibular first molar tooth germs of rats at were detected at embryonic day 15.5 (E15.5) (**A**), E17.5 (**B**), E19.5 (**C**), postnatal day 5 (P5) (**D–F**), P7 (**G–I**), P10 (**J–L**), P14 (**M–O**), P19 (**P–R**) and P25 (**S–U**). From E15.5-E19.5, Ape1 was intensely expressed in the mesenchymal cells including the dental papilla and dental follicle cells and in the dental epithelial cells including the inner and outer enamel epithelium (**A–C**). The expression of Ape1 became attenuated along with the maturity of DPCs which began to differentiate into odontoblast, and came to gather towards Hertwig’s epithelial root sheath (**D–R**). At P25, Ape1 expression was undetectable in the apical region (**U**), and the odontoblasts which were next to the newly formed pre-dentin were still positive for Ape1 expression at a relatively low level (**S,T**).

We collected rat mandible samples from E15.5 to P25 and examined the expression of Ape1 during tooth development by immunohistochemistry. Ape1 expression was found during the entire rat tooth germ developmental process and exhibited a nuclear localization. From E15.5 to E19.5 (Fig. 1A–C), Ape1 expression was detected intensely in the whole tooth germ both in the mesenchymal cells including the dental papilla and dental follicle cells and in the dental epithelial cells including the inner and outer enamel epithelium. It was also found in oral epithelium adjacent to the tooth. In the postnatal days from P5 to P25 (Fig. 1D–T), as the dental papilla cells began to differentiate into odontoblasts, Ape1 expression was rapidly decreased and gathered towards the Hertwig’s epithelial root sheath until the end of root formation. On P25, Ape1 was expressed in the odontoblasts next to the pre-dentin, but its expression was undetectable in the tooth apical region (Fig. 1U). The expression in the dental epithelial cells was also decreased along with the generation of enamel.

### Identification of DPCs and examination of Ape1 expression in DPCs

**Figure 2:**
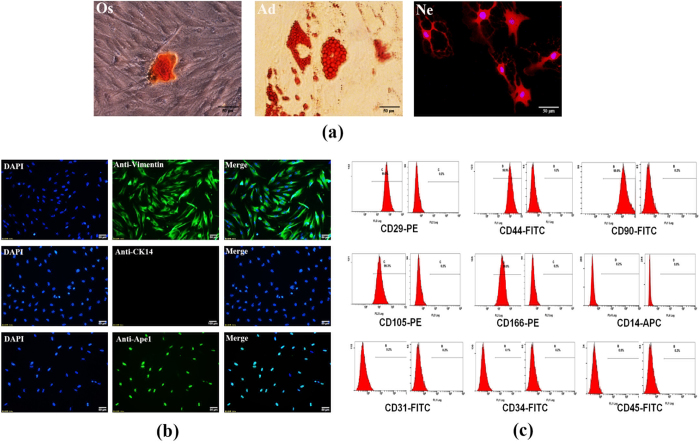
Characterization of DPCs: (**a**) After being separately cultured in osteogenic (Os) and adipogenic (Ad) media for 21 days, mineralized nodules were stained with alizarin red and oil red staining was used to assess the formation of oil droplets. DPCs which were cultured in neurogenic (Ne) media for 2 hours formed the axon-like structure. (**b**) Isolated DPCs were positive for Vimentin and negative for CK14 by immunocytochemistry; Ape1 was strongly expressed in DPCs, and were located in the nuclei at the same time; (**c**) Flow cytometric analysis revealed that cultured DPCs are positive for CD29 (99.8%), CD44 (99.9%), CD90 (99.9%), CD105 (99.3%) and CD166 (99.9%) but negative for CD14 (0.2%), CD31 (0.2%), CD34 (0.1%) and CD45 (0.0%). Mouse IgG isotype control antibodies conjugated to FITC, PE, or APC were used as negative controls. Scale bars: 50 μm.

To confirm and expand these *in vivo* observations especially in the dentin-formation process, we used DPCs, which are the progenitor cells of odontoblasts, to investigate potential function of Ape1 in odontogenesis. The multi-linage differentiation potential of DPCs was tested after being cultured in either osteogenic or adipogenic media for 21 days, and in neurogenic media for 2 hours. DPCs formed alizarin red stained mineralized nodules, oil-red–positive lipid droplet and processes formed extensive networks (Fig. 2a) respectively. Immunofluorescence assay (Fig. 2b) demonstrated that DPCs were stained positively for the mesenchymal cell marker vimentin, but negatively for epithelial cell marker cytokeratin. At the same time, immunofluorescence assay proved that Ape1 was detected in DPCs showing the nuclear localization. Similarly, there was a high expression level of MSC markers (i.e.CD29, CD44, CD90, CD105, and CD166), while the hematopoietic markers (i.e.CD34 and CD45), monocyte/macrophage marker (i.e.CD14) and megakaryocyte marker (i.e.CD31) were expressed at a very low level in DPCs as demonstrated by flow cytometry (Fig. 2c). These results revealed the stromal origin of these isolated cells with mesenchymal stem cell characteristics and the absence of hematopoietic and other precursor contamination.

### Elimination of Ape1 redox regulation stimulated osteo/odontogenic differentiation of DPCs

**Figure 3:**
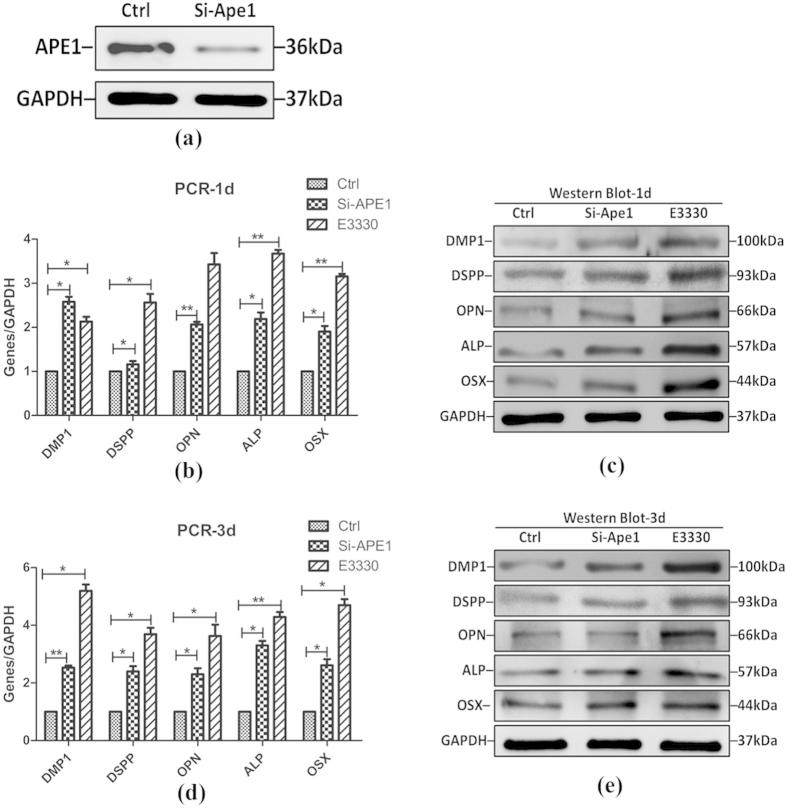
Ape1-ShRNA and E3330 enhanced osteo/odontogenic differentiation capacity of DPCs. (**a**) Western blot analysis demonstrates that Ape1-shRNA efficiently inhibits Ape1 gene expression, with 80–85% knockdown efficiency. (**b**) qRT-PCR analysis for odonto/osteogenic genes (*DMP1, DSPP, OPN, ALP* and *OSX*) in different groups at day 1. *GAPDH* served as an internal control. (**c**) Western blot analyses for the odonto/osteogenic proteins (DMP1, DSPP, OPN, ALP and OSX) in different groups at day 1. GAPDH served as an internal control. (**d**) qRT-PCR analysis for odonto/osteogenic genes (*DMP1, DSPP, OPN, ALP* and *OSX*) in different groups at day 3. *GAPDH* served as an internal control. (**e**) Western blot analyses for the odonto/osteogenic proteins (DMP1, DSPP, OPN, ALP and OSX) in different groups at day 3. GAPDH served as an internal control. **2−ΔΔCt ≥ 2, p < 0.01; *1 < 2−ΔΔCt < 2, p < 0.01; n = 3.

To investigate the function of Ape1 during DPCs differentiation, we applied Ape1-shRNA to inhibit Ape1 expression. Meanwhile, we used E3330 which was an Ape1 specific redox inhibitor in our research. It has been extensively proven that E3330 is able to accurately block the redox function of Ape1 without interfering with its DNA repair function^21^. Actually, we successfully established the Ape1-shRNA DPCs, and the Western blot analysis had proved that 80–85% of Ape1 was knocked down (Fig. 3a). As shown in Fig. 3b–e, Ape1-shRNA and E3330 treated groups remarkably increased the expression of osteo/odontogenic differentiation related genes and proteins (i.e.DMP1, DSPP, OPN, ALP and OSX), as examined by both qRT-PCR and Western blot analysis at day 1 and 3 in culture. However, as compared to E3330-treated group, Ape1-shRNA enhanced osteo/odontogenic differentiation capacity was relatively lower. Since the Ape1-shRNA and E3330 treated groups had the same influential tendency and E3330 appears to be much potent in stimulating osteo/odontogenic differentiation, we used E3330 in the subsequent studies.

### Ape1 redox inhibitor E3330 attenuated the proliferation of DPCs

**Figure 4:**
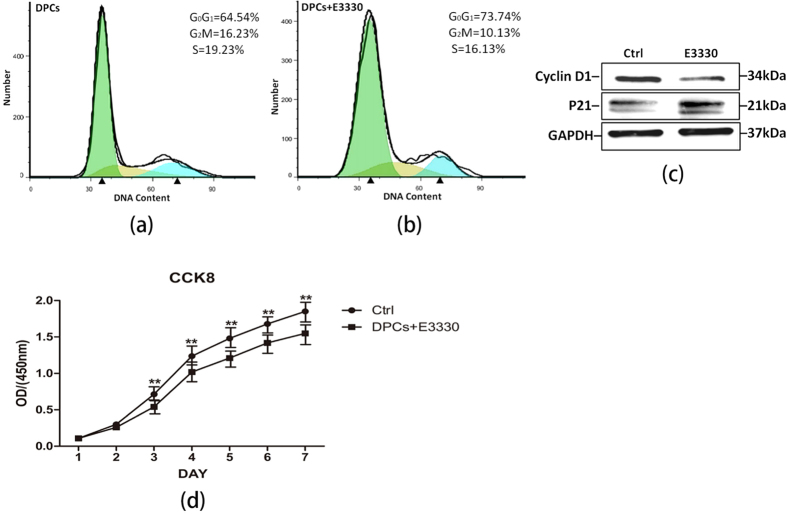
E3330 attenuates the proliferation of DPCs: (**a**) Representative cell-cycle distribution profiles of untreated DPCs at day 3. (**b**) Representative cell-cycle distribution profiles of E3330-treated DPCs at day 3. (**c**) Western blot analyses for cell cycle-related proteins Cyclin D1 and P21 in different groups at day 3. GAPDH served as an internal control. (**d**) Growth curves of untreated and treated DPCs plotted from CCK8 assay. The proliferation of E3330 treated DPCs (days 3–7) was attenuated compared with untreated ones (**P < 0.01).

Next, we focused on the cell proliferation, which was an indispensable part of stem cell biology. Cell-cycle analysis and CCK8 assay were performed to investigate whether E3330 could affect the proliferation of DPCs *in vitro*. DPCs obtained from different donors exhibited a similar downward trend of proliferation rate when treated by E3330. Flow cytometry was applied to investigate the proliferation index, i.e. the percentage of cells in S and G2M phases. As shown in Fig. 4a,b, the proliferation index in the E3330 treated group at day 3 (S + G2M = 26.26%) was dramatically lower than that in the untreated group (S + G2M = 35.46%), indicating that E3330 can attenuate the proliferation of DPCs. Western blot analysis (Fig. 4c) demonstrated that cell cycle positive related protein cyclin D1 was decreased and cell cycle negative related protein P21 was fortified in E3330-treated group, which was in accordance with the result from flow cytometry. Moreover, CCK8 assay (Fig. 4d) showed that the proliferation rate of E3330-treated groups were much lower as compared to that of untreated ones. OD values appeared significantly decreased in the E3330-treated DPCs compared with the untreated DPCs from day 3 to day 7 (P < 0.01).

### E3330 enhanced osteo/odontogenic differentiation of DPCs *in vitro*

**Figure 5:**
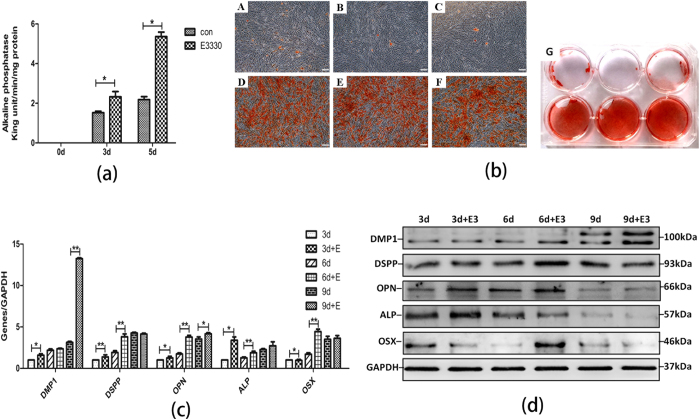
E3330 up-regulated osteo/odontogenic differentiation ability of DPCs. (**a**) Comparison of ALP activity between E3330 untreated and treated DPCs. Treated group presented a higher ALP activity at day 3 and day 5 than control group. (**b**) Comparison of the mineralized nodules betweenE3330 untreated and treated DPCs by alizarin red staining. E3330 treated DPCs generated more calcified nodules than untreated group after 14-day culture. Scale bars: 200 μm. (**c**) qRT-PCR analysis for odonto/osteogenic genes (*DMP1, DSPP, OPN, ALP* and *OSX*) in different groups at day 3, day 6 and day 9. *GAPDH* served as an internal control. (**d**) Western blot analyses for the odonto/osteogenic proteins (DMP1, DSPP, OPN, ALP and OSX) in different groups at day 3, day 6 and day 9. GAPDH served as an internal control. **2^−ΔΔCt^ ≥ 2, p < 0.01; *1 < 2^−ΔΔCt^ < 2, p < 0.01; n = 3.

To further evaluate the differentiation capability, alkaline phosphatase (ALP) assay and alizarin red staining were applied to evaluate capacity of mineralization at defined time points. ALP activity of DPCs was increased by E3330 at day 3 and 5 (Fig. 5a). Alizarin red staining at Day 14 showed the presence of small, mineralized nodules in control group in osteogenic media, whereas the E3330-treated group displayed a greater number of nodules, which were fused with other to form larger mineralized nodules (Fig. 5b). Quantitative RT-PCR (Fig. 5c) was performed to evaluate the expression levels of osteo/odontogenic genes including *DMP1, DSPP, OPN, ALP* and *OSX*. Compared with the untreated group, there was a remarkable increase of the osteogenic markers (i.e. *DMP1, OPN, ALP* and *OSX*.) at days 6 and 9 in E3330 treated DPCs. At the same time, *DSPP* mRNA level was mildly up-regulated at day 3, and then overwhelmingly increased at day 6 in treated DPCs. The observed expression changes were confirmed on protein level by Western blot (Fig. 5d). The protein expression of DMP1 (at days 6 and 9), OSX (at days 6 and 9), DSPP (at days 3, 6 and 9), OPN (at days 3 and 6) and ALP (at days 3 and 6) in the E3330 treated group was elevated accordingly.

### E3330 enhances DPCs osteo/odontogenic differentiation via canonical Wnt signaling pathway

**Figure 6:**
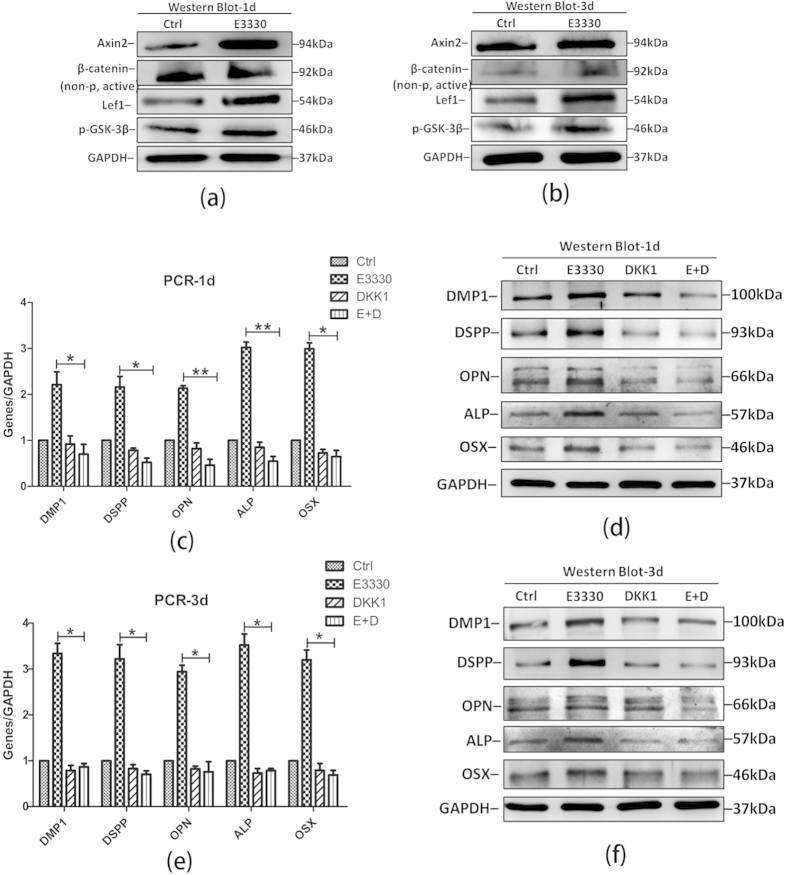
E3330 enhances DPCs osteo/odontogenic differentiation via canonical Wnt signaling pathway. (**a**) Western blot analyses for the canonical Wnt signaling pathway related proteins (Axin2, non-p active β-catenin, Lef1 and p-GSK-3β) in different groups at day 1. GAPDH served as an internal control. (**b**) Western blot analyses for the canonical Wnt signaling pathway related proteins (Axin2, non-p active β-catenin, Lef1 and p-GSK-3β) in different groups at day 3. GAPDH served as an internal control. (**c**) qRT-PCR analysis for odonto/osteogenic genes (*DMP1, ALP, DSPP, OSX* and *OPN*) in addition with rhDKK1 in different groups at day 1. *GAPDH* served as an internal control. (**d**) Western blot analyses for osteo/odontogenic related proteins (DMP1, ALP, DSPP, OSX and OPN) in addition with rhDKK1 in different groups at day 1. GAPDH served as an internal control. (**e**) qRT-PCR analysis for odonto/osteogenic genes (*DMP1, ALP, DSPP, OSX* and *OPN*) in the presence of rhDKK1 in different groups at day 3. *GAPDH* served as an internal control. (**f**) Western blot analyses for osteo/odontogenic related proteins (DMP1, ALP, DSPP, OSX and OPN) in addition with rhDKK1 in different groups at day 3. GAPDH served as an internal control. **2^−ΔΔCt^ ≥ 2, p < 0.01; *1 < 2^−ΔΔCt^ < 2, p < 0.01; n = 3. E + D, E3330 + rhDKK1.

Wnt signaling pathway has been previously shown to play important roles in dentinogenesis. Therefore, we further evaluated the role of Wnt/β-catenin signaling in E3330-induced osteo/odontogenesis of DPCs. At day 1 and day 3 (Fig. 6a,b), the expression of Axin2, Lef1, non-phospho (active) β-catenin (Ser33/37/Thr41) and p-GSK-3β (Ser9) was abundantly increased in the E3330-treated groups, indicating the activation of canonical Wnt signaling. We then applied rhDKK1 which was a potent antagonist of canonical Wnt signaling pathway to verify whether the canonical Wnt signaling pathway is involved in the DPCs osteo/odontogenic differentiation induced by E3330. Our results showed that in the presence of rhDKK1, E3330-stimulated expression of osteo/odontogenic differentiation related genes and proteins was significantly reduced (Fig. 6c–f), suggesting that E3330 functions through canonical Wnt signaling pathway to activate osteo/odontogenic differentiation in DPCs.

## Discussion

In present study, for the first time we described the inhibition of Ape1 redox activity promoted the osteo/odontogenic differentiation capacity of DPCs via canonical Wnt signaling pathway. DPCs are a novel stem cell population that was identified in the dental mesenchyme of the third molar tooth germ during the crown-forming stage^10^. Once DPCs contact predentin which is produced by perodontoblasts, they begin to differentiate into odontoblasts that secret the extracellular matrix and gradually move backward, and this process is highly organized by multiple interactions between signaling pathways and cytokines^26^. The disturbance of the dentin originating during the histodifferentiation stage of tooth development will give rise to dentinogenesis imperfecta which exhibits the tooth discoloration and enamel fracture due to poorly mineralized dentin^27^. As a transcriptional coactivator, Ape1 plays a crucial role in controlling different cellular processes such as differentiation, proliferation and apoptosis by both redox-dependent and -independent mechanism^28^,^29^. Currently, direct evidence of the role of Ape1 in regulating dentinogenesis is lacking. Therefore, it is the first time that we introduce Ape1, especially its function of redox regulation, into dental tissue-derived stem cells differentiation and proliferation. As is expected, the above observations in this study are in line with some previous studies reporting that the inhibition of Ape1 can promote the differentiation process and attenuate the proliferative ability in cardiac stem cells and neurogenic embryonic cell^24^,^25^. In contrast, Ape1 positively regulates hematopoietic differentiation of embryonic stem cells through its redox functional domain^30^. This discrepancy may be attributed to the different cell populations used and distinct cell microenvironments (niches) cultured.

In this study, we showed that Ape1 is expressed in the developing tooth throughout the entire developmental process, and its expression decreased gradually from E15.5 to P25. Our results also suggest that the dual function of Ape1 may play a complicated role in tooth development: on the one hand, Ape1 is indispensable during this process, because Ape1 is one of the key enzymes of the base excision repair (BER) pathway which happens per cell per day depending on the tissue type in mammals^31^,^32^. On the other hand, the attenuated expression of Ape1 suggests that disappearance or disruption of Ape1 expression is associated with odontogenic differentiation at the late developmental stages. In fact, protein down-regulation is always associated with a decrease in both redox and AP endonuclease activity^33^. This speculation is supported by the *in vitro* experiments in which we apply E3330 and Ape1-shRNA to block or eliminate the part or whole function of Ape1, and show the promotion of osteo/odontogenic differentiation of dental stem cells.

Ape1 appears to affect the proliferation of DPCs through its redox regulation, as evidenced by the fact that the proliferation of DPCs was significantly crippled from day 3 and cyclin D1 was down-regulated when treated with E3330. However, the mechanistic details underpinning Ape1-regulated proliferation in DPCs are currently unknown and require future investigation. In pancreatic cancer cells, E3330 treatment resulted in reduced level of cyclin D1 that is controlled by NF-κB signaling^34^. In lung cancer cells CL3, Ape1 redox activity facilitated the cyclin D1 expression and G1 to S progression following ERK activation^35^. Even in human keratinocytes inflammatory responses, silencing of Ape1 attenuated cyclin D1 expression and phosphorylation of ERK1/2 and Akt, thereby affecting its proliferation^36^. As a redox-modifying factor, Ape1 can stimulate numerous transcription factors and some of them have a positive relationship with cell proliferation. Therefore, in our current study, the inhibition of cell proliferation in DPCs by E3330 can be attributed, at least partially, to the reduction in cyclin D1 level.

Inhibition of Ape1 redox activity promoted the osteo/odontogenic differentiation capacity in DPCs. Several osteo/odontogenic-related markers such as DMP1, DSPP, OPN, ALP and OSX are up-regulated when exposed to E3330 and Ape1-shRNA treatment. It is widely recognized that DMP1 is a non-collagenous calcium-binding protein that plays a critical role in biomineralization and has been implicated in signal transduction in the process of odontogenesis^37^. As to DSPP/DSP, it is a well-known marker of odontoblasts and highly expressed in dentin or predentin and essential for dentinogenesis^38^. DSPP can also control the hydroxyapatite mineral phase during dentin calcification^39^. OPN is a secretory calcium-binding phosphorylated glycoprotein that plays an important role in bone remolding, which is also considered as a principle component of bone^40^. As a characteristic marker of osteoblast phenotype, ALP is an important marker during the early stage of bone formation^41^,^42^. *OSX* acts as a downstream gene of RUNX2 that is highly expressed in the functional osteo/odontoblasts^43^,^44^. Those genes and their transcripted proteins play a vital role in dentin formation and tooth engineering, and a suitable microenviroment created by E3330 guarantees the high-efficiency expression of them.

It is generally accepted that several conserved signaling pathways, including BMP, Wnt, FGF and Notch pathways, play important roles throughout tooth development^45^. Much of the researches have widely proved that canonical Wnt signaling is essential in activating odontogenic mesenchyme during tooth development^46^. In our current studies, E3330 treatment enhances the ability of osteo/odontogenic differentiation of DPCs through canonical Wnt signaling pathway, and the addition of DKK1 which is a potent antagonist of canonical Wnt signaling pathway blocks this activation. It’s the first time that we demonstrate a relationship between Ape1 and canonical Wnt signaling pathway during tooth development. However, the relationship between Ape1 and Wnt signaling pathway warrants further investigation. Dai *et al* have reported that Ape1 contributes to the Wnt signaling pathway by using KEGG pathway annotation, and Wnt signaling pathway is abundant when exposed to up-regulated mi-RNA in Ape1 knockdown HOS cells^47^. Except that, the existing studies which link the canonical Wnt signaling pathway and Ape1 are very rare. Noteworthily, recent studies have addressed the important role of Ape1 in the regulation of stem cell physiology in a ROS-related manner. As a redox sensor, Ape1 monitors intracellular ROS level by modulating the activity of several transcription factors including HIF-1 α, p53 and NF-κB^48^, or inhibiting Rac1-regulated membrane-bound NADPH oxidase (NOX) to regulate ROS production^49^. The ROS level mediates the self-renewal and differentiation of stem cells and a numerous of observations have confirmed that low levels of ROS maintain ‘stemness’, whereas higher levels of ROS promote differentiation in different types of stem cells^50^,^51^. Interestingly, canonical Wnt signaling pathway can be triggered by endogenous ROS in a β-catenin dependent way. The augmented endogenous ROS level releases the redox-sensitive binding sites, hence promoting a DVL-mediated stimulation of the downstream of WNT/β-catenin signal transduction^52^. In immortalized neural progenitor cell line, mitochondrial Ca^2+^ influx stimulates endogenous ROS production and mediates Wnt/β-catenin pathway activity to facilitate cell differentiation^53^. Except being mediated by endogenous ROS, Wnt/β-catenin pathway can be blocked by exogenous ROS induced by some metabolic diseases such as diabetes and tumor^54^,^55^. In our research, we don’t set foot in this ROS-mediated field because we have to identify the work Ape1 actually does during tooth development first, so we can go deep inside understanding the role Ape1 plays next. In stomatology, there are many oral diseases like odontogenic tumor and clinical therapies especially orthodontics treatment connecting with redox status. Thus, our findings not only bring new perspective for thoroughly understanding the osteo/odontogenic differentiation ability of DPCs, but also contribute to lay a solid foundation for further researches which focused on redox-regulated tooth development and regeneration.

**Figure 7:**
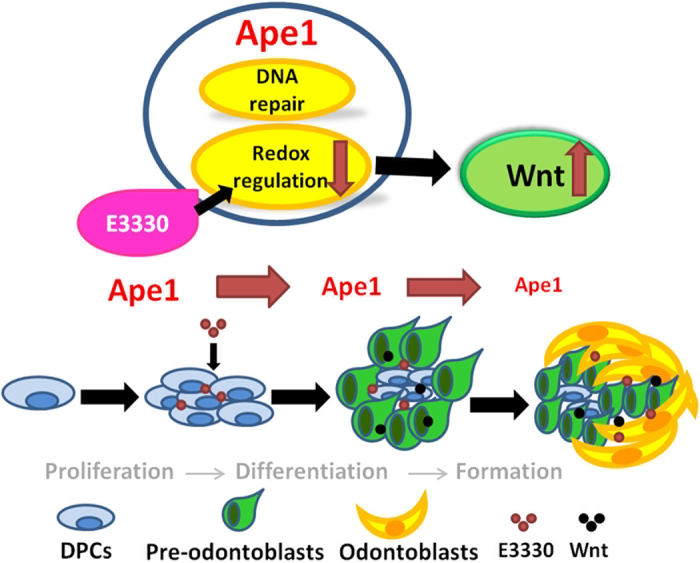
Schematic illustration of the function of DPCs in response to treatment of E3330. The expression of Ape1 is attenuated during the maturation of DPCs. The existence of Ape1 is necessary for DPCs proliferation but may hinder the differentiation of DPCs. E3330 blocks the redox regulation which is one of two main functions (another one is DNA repair function) of Ape1, and enhances DPCs osteo/odontogenic differentiation capability by activating canonical Wnt signaling pathway.

In conclusion, the *in vitro* and *in vivo* evidence accumulated in the present study revealed for the first time that the inhibition of Ape1 redox activity can promote the osteo/odontogenic differentiation via canonical Wnt signaling pathway in DPCs (Fig. 7). These findings in DPCs implicate that inhibition of Ape1 redox activity with E3330 could be a promising therapeutic strategy for tooth tissue engineering. Further studies are required to investigate the ROS-related mechanism during E3330 enhanced osteo/odontogenic differentiation in DPCs.

## Methods

All experiments were conducted in accordance with the ethical protocol approved by the Committee of Ethics of the Sichuan University. In addition, for investigations involving human subjects, informed consent has been obtained from the participants involved. All the methods were carried out in accordance with the approved guidelines.

### Cell culture and identification

According to the patients’ panoramic radiographs, tooth germs of the impacted third molar at the crown-forming stage were collected from patients (n = 12, 12–16 years of age) with written consent signed by parents during orthodontic treatment in the West China Stomatology Hospital. DPCs were derived as previously described^8^. Briefly, The dental papilla tissues were carefully isolated using a dental tweezer under the stereomicroscope (Leica2000,Zeiss, Jena, Germany), minced into about 1*1 mm^3^ pieces and digested in a solution containing 3 mg/ml collagenase type I (Sigma, St. Louis, MO) and 4 mg/ml dispase (Sigma, St. Louis, MO) for 20 min at 37 °C. The samples were then cultured in alpha-Modified Eagle’s Medium (α-MEM; Hyclone, USA) supplemented with 15% FBS (Hyclone, USA), 1% P/S (Solarbio, Beijing, China). The fresh medium was changed every 2 days. Cells were subcultured at the ratio of 1:3 when they reached 80~85% confluence. DPCs at 3–4 passages were used for subsequent experiments.

To determine the nature of cultured cells, isolated cells at passage 3 (P3) were immunofluorescenced with the antibody against vemintin (1:400, Thermo, USA) and cytokeratin (1:400; Abcam, USA). Subsequent steps were performed according to the manufacturer’s recommendations, and all samples were examined under a fluorescence microscope (Leica DMI 6000, Germany). Flow cytometric analysis of specific surface antigens was also used to characterize the cultured cells. Cells were harvested and incubated with various combinations of the following florochrome-conjugated mouse anti-human antibodies: CD14-APC, CD29-PE, CD31-FITC, CD34-FITC, CD44-FITC, CD45-FITC, CD90-FITC, CD105-PE and CD166-PE (all from BD Biosciences, USA) for 20 min at room temperature in the dark. The corresponding mouse IgG isotype control antibodies conjugated to FITC, PE and APC were employed as negative controls in each experiment. Flow-cytometry was carried out using the Beckman Coulter Cytomics FC 500 MPL system (Beckman Coulter, USA).

### Multipotential differentiation of DPCs

For detecting of multipotential differentiation capacity of DPCs, a total of 1 × 10^5^ DPCs were seeded into each well of a six-well plate. At 60–70% confluence, DPCs were cultured in osteogenic medium (α-MEM supplemented with 10% FBS, 5 mM L-glycerophosphate, 100 nM dexamethasone, and 50 μg/mL ascorbic acid) and in adipogenic medium (α-MEM supplemented with 10% FBS, 2 mM glutamine, 100 U/mL penicillin streptomycin, 100 μM ascorbic acid, 0.5 mM isobutylmethylxanthine, 0.5 Mm hydrocortisone and 60 mM indomethacin) for 21 days, respectively. DPCs for osteogenic differentiation was incubated in 0.1% Alizarin Red S solution (pH = 4.3; Sigma-Aldrich, USA) at room temperature for 30 min, and for adipogenic differentiation one incubated in 0.3% Oil Red O (Sigma-Aldrich, USA) solution for 15 min. After washing three times in PBS, cells were routinely observed and visualized under a light microscope. For neurogenic differentiation, cells were exposed to neurogenic medium consisted of α-MEM supplemented with 10% FBS, 2 mM glutamine, 100 U/mL penicillin streptomycin, 2% dimethylsulphoxide, 200 μM butylated hydroxyanisole, 25 mM KCl, 2 mM valproic acid sodium salt, 10 mM forskolin, 1 mM hydrocortisone and 5 μg/ml insulin for 2 hours. After fixed in 4% paraformaldehyde for 15 min, the samples were immunofluorescenced with the antibody against β III-tubulin (1:200; Abcam, USA). Subsequent steps were performed according to the manufacturer’s recommendations and examined under a fluorescence microscope (Leica DMI 6000, Germany).

### Immunohistochemistry

In order to examine the expression of Ape1 during rat tooth germ developmental process, we collected rat mandibular samples from E15.5 to P25 for a continuous observation. Immunohistochemical analyses of the samples were performed using the streptavidin-biotin complex method according to the manufacturer’s recommended protocol. Briefly, tissue sections (4 μm) from representative paraffin blocks were deparaffinized in xylene and rehydrated through graded ethanol solutions. Endogenous peroxidases were blocked using 3% hydrogen peroxide. For the antigen retrieval, the sections were processed by the conventional microwave heating in 0.01 M sodium citrate retrieval buffer (0.01 M sodium citrate and 0.01 M citric acid, pH 6.0) for 30 min. Then, the sections were blocked by 10% normal goat serum for 30 min at 37 °C and incubated with primary antibodies (Ape1, 1:200 abcam, USA) overnight at 4 °C. Finally, the sections were incubated with secondary antibodies for 1 h at room temperature. The sections were then stained using a 3,3′-diaminobenzidine DAB kit. The immune reactions were visualized under a light microscope (Olympus BX43F; JEOL, Tokyo, Japan).

### Treatment of DPCs with E3330 or DKK1

E3330 [(2E)-3-[5-2, 3-dimethoxy-6-methyl-1,4-benzoquinoyl)]-2-nonyl-2-propenoicacid] (Sigma-Aldrich, USA) was first dissolved in DMSO, at a concentration of 10 Mm. A dose of 10 μM of E3330 was applied throughout this study according to our observation that 10 μM E3330 significantly increased differentiation capacity of DPCs in the absence of dose-related adverse effects. A vehicle control with matched concentration of DMSO without E3330 was included in each experiment. Recombinant human rat DKK1 proteins were purchased from R&D (Wiesbaden, Germany). For preparation of stock solutions, proteins were dissolved in PBS containing 0.1% bovine serum albumin (BSA). DPCs were treated with 10 μM E3330, 100 ng/mL DKK1^56^ and E3330 + DKK1 respectively.

### Transfection with lentivirus

DPCs from Passage 3(P3) were seeded to 6-well plates at 1 × 10^6^ cells/well with fresh culture medium containing no antibiotics and fetal bovine serum. For experiments, cells were infected with lentivirus expressing shRNA specific to Ape1 mRNA coding sequence. Transfection reagent was removed after 8 h and the transfected cells were selected by applying puromycin (400 ng/ml) in culture medium until the cells in control group were all died. The stable Ape1-shRNA and scrambled-Ape1 cell lines were obtained.

### CCK8 assay for cell growth

CCK8 assay was performed to investigate the cell proliferation. Briefly, cells were plated in flat-bottomed 96-well microplates at 2 × 10^3^ cells/well, and divided into E3330 treated and untreated groups. After 1, 2, 3, 4, 5, 6 and 7 days in culture, 10 *μ*L/well fresh CCK8 solution (Dojindo, Japan) was added to the wells at the end of the experiment. After incubation at 37 °C for 1.5 hours, the absorbance of each well was determined using a microplate reader at 450 nm. The degree of cell proliferation was determined as the percentage of absorbance of treated cells to that of control cells. Five independent experiments were performed in each group, and the graph was plotted according to the average value.

### Flow cytometry for cell cycle

After 3-day in culture, E3330 treated and untreated DPCs (1 × 10^6^) were respectively collected by exposure to trypsin/EDTA for 1 min and centrifuged at 1000 rpm for 5 min. Cell precipitates were washed twice with 0.01 mol/L PBS containing 2% FBS and resuspended in 1 mL physiologic saline, fixed in 500 *μ*L cold 75% alcohol, and stored at 4 °C overnight. Then, each sample was washed again with PBS, and incubated with RNase at 37 °C for 30 min and then added with propidium iodide (100 mg/mL; Keygentec, Nanjing, China) at 4 °C for at least 30 min. Cell cycle fractions (G0G1-, S-, G2M-phases) were then determined by Beckman Coulter Cytomics FC 500 MPL system (Beckman Coulter, USA). Three independent experiments from different donors were performed for each group.

### Alkaline phosphatase (ALP) assay and alizarin red staining

DPCs were grown in osteogenic medium containing 10 μM E3330. At days 3 and 5, ALP activity of E3330 treated and untreated DPCs was determined according to the manufacturer’s recommendations with an ALP kit (Jiancheng, Nanjing, China) and normalized on the basis of equivalent protein concentrations. The absorbance of each well was determined using a microplate reader at 520 nm. Calcium deposition in the extracellular matrix was determined by Alizarin Red S staining after 14 days of osteogenic differentiation. Cells were fixed in 4% polyoxymethylene (Sigma-Aldrich, USA) and then incubated in 0.1% Alizarin Red S solution (pH = 4.3; Sigma-Aldrich, USA). Calcium deposition was observed and visualized under a light microscope, and the picture of 6-wells plate was captured by camera.

### Quantitative RT-PCR

Total cellular RNA was harvested by adding TRIzol reagent (Invitrogen, Carlsbad, USA) to cell samples. Isolated RNA precipitates were completely dissolved in diethypyrocarbonate(DEPC) treated water (Ambion Inc., Austin, USA) and reversely transcribed using SuperScript® III cDNA Synthesis Kit (Invitrogen, USA). Quantitative RT-PCR was performed using SYBR® Premix Ex Taq™ kit (TaKaRa, Bio, Otsu, Japan) and ABI7300 Real-Time PCR System (Applied Biosystems, USA). Primer sets used for the detection of DMP1, DSPP, OPN, ALP, OSX and GAPDH were listed in Table 1. Quantitative RT-PCR reaction conditions were: 95 °C for 30 s; followed by 40 cycles of 95 °C for 5 s, 60 °C for 3o s; ended with 95 °C for 15 s,60 °C for 60 s, 95 °C for 15 s. The results were calculated from three independent experiments.

### Western blot analysis

DPCs were isolated by differential digestion as described above, after which total protein was extracted and normalized according to the manufacturer’s instructions. The primary antibodies were anti-APE1 (1:1,000; abcam, USA), anti-DMP1 (1:1,000; Santa cruz, USA), anti-DSP1-H (1:1,000; Santa cruz, USA), anti-OPN (1:1000; abcam, USA), anti-ALP (1:1,000; abcam, USA), anti-OSX (1:1,000; abcam, USA), anti-Axin (1:1000; abcam, USA), anti-Lef1 (1:1000; abcam, USA), anti-non-p (active) β-catenin (1:1000; CST, USA), anti-p-GSK-3β (1:1000; CST, USA), anti-P21 (1:1000; abcam, USA), anti-cyclin-D1 (1:1,000; Santa cruz, USA) and anti-GAPDH (1:10,000; Zen, China) used as internal control. Then, the membranes were rinsed with TBST (0.1% Tween-20 in 0.01 mol/L TBS), incubated with appropriate horseradish peroxidase conjugated secondary antibodies at 1:5000 (Santa Cruz, USA) at room temperature for additional 2 h, visualized by Image Quant LAS 4000 mini (GE, UK). Densitometry analysis on the bands was performed using the NIH image J software and normalizing the data to total protein levels (Supplementary Fig. 1. and Supplementary Fig. 2).

### Statistics analysis

The quantitative results were expressed as mean ± SD. Independent samples t-test and Chi-square test were performed with SPSS-Windows v.14.0 software (SPSS, USA). P-values less than 0.05 were considered to be statistically significant.

## Additional Information

**How to cite this article**: Chen, T. *et al.* Inhibition of Ape1 Redox Activity Promotes Odonto/osteogenic Differentiation of Dental Papilla Cells. *Sci. Rep.* **5**, 17483; doi: 10.1038/srep17483 (2015).

## Supplementary Material

Supplementary Information

## Figures and Tables

**Table 1:** Forward and reverse primers for reverse transcription-polymerase chain reaction.

Gene	GenBank No.	Sequences (5′-3′)	Size
*DMP1*	NM_004407.3	Forward: CTCGCACACACTCTCCCACTCAAA	180
		Reverse: TGGCTTTCCTCGCTCTGACTCTCT	
*DSPP*	NM_014208.3	Forward: CTGTTGGGAAGAGCCAAGATAAG	129
		Reverse: CCAAGATCATTCCATGTTGTCCT	
*OPN*	NM_001040058.1	Forward: CAGTTGTCCCCACAGTAGACAC	127
		Reverse: GTGATGTCCTCGTCTGTAGCATC	
*ALP*	NM_000478.4	Forward: TAAGGACATCGCCTACCAGCTC	170
		Reverse: TCTTCCAGGTGTCAACGAGGT	
*OSX*	NM_001173467.2	Forward: GAGGTTCACTCGTTCGGATG	120
		Reverse: TGGTGTTTGCTCAGGTGGT	
*GAPDH*	NM_002046.5	Forward: CTTTGGTATCGTGGAAGGACTC	132
		Reverse: GTAGAGGCAGGGATGATGTTCT	
