# Supplementary material for: Inhibition of Ape1 Redox Activity Promotes Odonto/osteogenic Differentiation of Dental Papilla Cells
Source: Sci Rep. 2015 Dec 7;5:17483. doi: 10.1038/srep17483 (PMC4671010; doi:10.1038/srep17483)
Supplement: Supplementary Information [file srep17483-s1.pdf]

Inhibition of Ape1 Redox Activity Promotes Odonto/osteogenic Differentiation of Dental Papilla Cells

Tian Chen[1,2,3], Zhi Liu [1,2], Wenhua Sun[1,2], Jingyu Li[1,2,3], Yan Liang[1,2], Xianrui Yang[1,2,3], Yang Xu[1,2,3], Mei Yu[1,2], Weidong Tian[1,2,4], Guoqing Chen[1,2,*] and Ding Bai[1,2,3*]

1National Engineering Laboratory for Oral Regenerative Medicine, West China Hospital of Stomatology, Sichuan University, Chengdu 610041, P.R. China

2State Key Laboratory of Oral Diseases, West China Hospital of Stomatology, Sichuan University, Chengdu 610041, P.R. China

3Department of Orthodontics, West China College of Stomatology, Sichuan University, Chengdu 610041, P.R. China

4Department of Oral and Maxillofacial Surgery, West China College of Stomatology, Sichuan University, Chengdu 610041, P.R. China

*Correspondence to: Guoqing Chen, National Engineering Laboratory for Oral Regenerative Medicine, West China Hospital of Stomatology, Sichuan University, No.14, 3rd Section, Renmin South Road, Chengdu 610041, PR China. Tel/fax: +86 28 8550 3499. E-mail address: chen21gq@163.com

Ding Bai, Department of Orthodontics, West China Hospital of Stomatology, Sichuan University, No.14, 3rd Section, Renmin South Road Chengdu 610041, PR China. Tel/fax: +86 28 8550 3499. E-mail address: baiding@ scu.edu.cn

Supplementary Figures

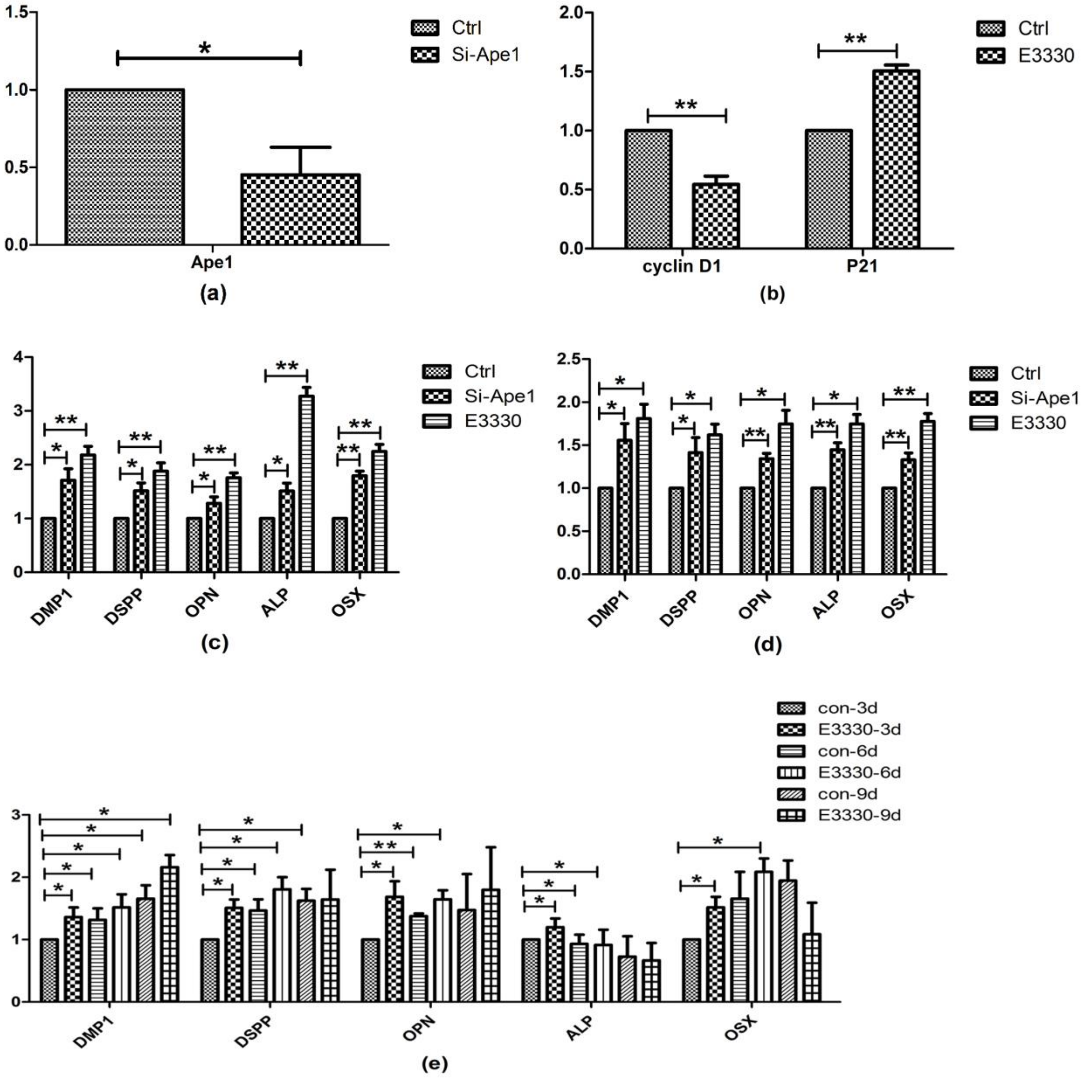

Supplementary Fig 1. Densitometry analysis on the western blotting bands. (a) Quantification of western blotting data of Fig 3a. (b) Quantification of western blotting data of Fig 4c. (c) Quantification of western blotting data of Fig 3c. (d) Quantification of western blotting data of Fig 3e. (e) Quantification of western blotting data of Fig 5d. Densitometry analysis on the bands was performed using the NIH image J software and normalizing the data to total protein levels. GAPDH served as an internal control. *P <0.05, ** P < 0.01, n= 3.

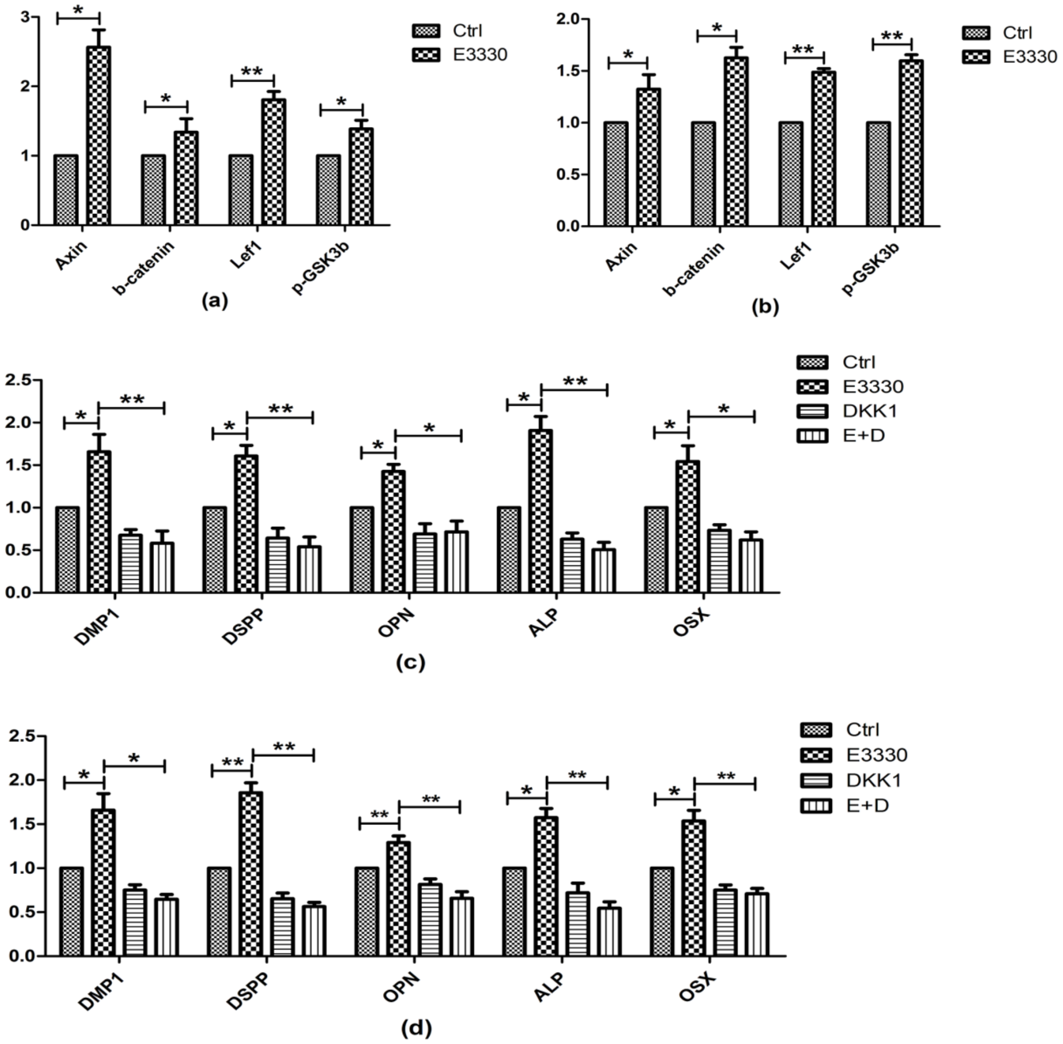

Supplementary Fig 2. Densitometry analysis on the western blotting bands. (a) Quantification of western blotting data of Fig 6a. (b) Quantification of western blotting data of Fig 6b. (c) Quantification of western blotting data of Fig 6d. (d) Quantification of western blotting data of Fig 6f. Densitometry analysis on the bands was performed using the NIH image J software and normalizing the data to total protein levels. GAPDH served as an internal control. *P < 0.05, ** P < 0.01, n= 3.
